# Effect of active application of self-etching ceramic primer on the long-term bond strength of different dental CAD/CAM materials

**DOI:** 10.4317/jced.58723

**Published:** 2021-11-01

**Authors:** João-Paulo-Mendes Tribst, Pedro-Jacy-Santos Diamantino, Maiara-Rodrigues de Freitas, Isabela-Vitelli Tanaka, Lais-Regiane Silva-Concílio, Renata-Marques de Melo, Guilherme-de Siqueira-Ferreira-Anzaloni Saavedra

**Affiliations:** 1 DDS, MSc, PhD, Department of Dentistry, University of Taubaté (UNITAU), Taubaté 12020-270, SP, Brazil; 2 DDs, MSc, PhD student, Department of Dental Materials and Prosthodontics, São Paulo State University (Unesp), Institute of Science and Technology, São José dos Campos, São Paulo, Brazil; 3 DDS, MSc, PhD student, Department of Dentistry, University of Taubaté (UNITAU), Taubaté 12020-270, SP, Brazil; 4 DDs, MSc, PhD, Department of Dental Materials and Prosthodontics, São Paulo State University (Unesp), Institute of Science and Technology, São José dos Campos, São Paulo, Brazil

## Abstract

**Background:**

The objective of this in vitro study was to evaluate the effect of the active application of self-etching ceramic primer (ME&P) on the bond strength of different dental CAD/CAM materials (Lithium Disilicate ceramic (LD), Leucite ceramic (LE), Zirconia reinforced lithium silicate ceramic (ZLS), and Hybrid ceramic (HC)) with thermocycling aging.

**Material and Methods:**

The samples were randomly divided into 16 groups (n = 20). Dual resin cement cylinders were made and light cured for 10 s (1.200 mW/cm2) for the shear bond strength test. 3-way ANOVA revealed that the factors were statistically significant (*P*< 0.05).

**Results:**

The aging process had a negative impact on the bond strength for all groups except for Lithium Disilicate, with active application. ZLS and LE showed promising results with high bond strength values for the ME&P active application; however, after aging the bond strength value was significantly reduced. HC showed reduced bond strength values regardless the ME&P application.

**Conclusions:**

In order to obtain a durable bond strength, the recommended protocol of 20 s of active application followed by 40 s of sitting time in the self-etching ceramic primer should be followed when using reinforced-glass ceramics as restorative materials.

** Key words:**Dentistry, dental materials, silane, shear strength, computer-aided design

## Introduction

With the main objective of preserving the remaining dental structures and to recover masticatory function and aesthetics, the dental ceramics are widely applied as biomaterials in Dentistry (1). In addition to being biocompatible, these materials can mimics the optical characteristics of teeth in a satisfactory way (2), present good mechanical properties and long-term color stability (3,4). However, dental ceramics strength is usually inversely proportional to the aesthetics (5,6).

Nowadays, clinicians and researchers must know the available dental materials that can be indicated to make the restorative procedure more predictable and safer for the patients. Therefore, reinforced glass ceramics (Leucite, Lithium disilicate and Zirconia reinforced lithium silicate based materials), as well as hybrid ceramics (polymer infiltrated ceramic network) are versatile materials that combine proper aesthetic and mechanical properties to manufacture crowns, partial restorations (inlays and onlays) and implant-supported restorations (7). These materials can only be applied in dentistry due to the advancement of CAD/CAM (Computer aided design and computer aided manufacturing) technology, which allows the machining of indirect restorations with excellent structural characteristics.

Despite the restorative material selection, for a predictable treatment, the adhesive procedure should be properly performed aiming to achieve the most durable bond strength in long-term follow-ups. Therefore, the restorative material should receive a surface treatment that will improve the bondability with the resin cement. Conventionally, surface treatment is performed using hydrofluoric acid (HF) etching, which creates mechanical retentions and makes the surface more reactive followed by the application of silane coupling agent, whose function is to unite the inorganic portion of the ceramic with the organic portion of the resin cement (8-10). However, in order to simplify the process and to reduce the use of hazardous HF, using a self-etching ceramic primer (Monobond Etch & Prime, Ivoclar Vivadent, Schaan, Liechtenstein) is an excellent alternative.

The principal advantage of self-etching ceramic primer (ME&P) usage is the decrease in the number of clinical steps. This makes the procedure less sensitive to the technique. In addition, by having a single exposure time for all ceramic materials, the ME&P allows standardization, which contributes to the simplicity of the luting procedure (10,11). The manufacturer recommendation is to apply the ME&P on the ceramic surface actively for 20 seconds with the aid of a microbrush and then let sitting for 40 seconds. Until now, however, there is no report in the literature that justifies if this actively application presents any beneficial effect to the bond strength or if 60 seconds of exposure timing could be performed as a simpler process.

It is also important to evaluate the bond strength of dental materials in long-term simulations, performing aging procedures. This is important since the oral environment is an unfriendly medium to the restorative materials, which will stress the adhesive interface, impair the bond strength and reduce the restoration survival as long it remains in position. A valuable method, widely applied in literature, is the use of thermocycling aging which mimics some of the aspects of the oral cavity temperature variation (12).

Therefore, the aim of the present study was to evaluate the effect of the active application of a self-etching ceramic primer on the bond strength of different CAD/CAM materials before and after aging simulation. The null hypothesis was that the different surface treatment would not affect the bond strength regardless the restorative materials and aging process.

## Material and Methods

-Sample preparation

Three hundred twenty (320) blocks of four different CAD/CAM materials (Lithium Disilicate based ceramic (LD), Leucite based ceramic (LE), Zirconia reinforced lithium silicate based ceramic (ZLS), and Hybrid ceramic (HC)) were cut with a low-speed diamond saw using water cooling into 6 x 6 x 2 mm blocks verified with the aid of a digital caliper (Eccofer, Curitiba, PR, Brazil). The block surfaces were flattened with decreasing granulated SIC paper (#600, #800, #1000 and #1200 - 3M, St. Paul, MN, USA) using an automatic polishing machine (Ecomet 250 Grinder Polisher, Buehler, Illinois, USA). The lithium (di)silicate samples were subsequently crystalized in a ceramic furnace of the respective ceramic systems according to the manufacturer’s recommendations. The materials’ respective manufacturers and compositions are summarized in Table 1.

**Table 1:**
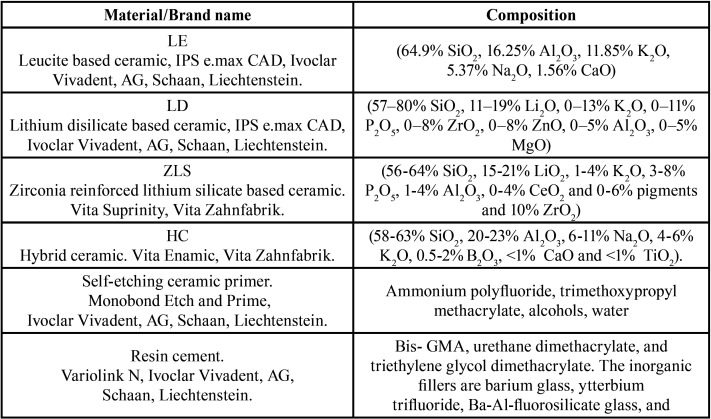
Materials used in this study and their respective manufacturers and compositions.

The 320 blocks were embedded in chemically activated acrylic resin (TDV, TDV Dental Ltda, Pomerode, Santa Catarina, Brazil) using a polyvinylchloride cylinders mold. Then, the samples were randomly divided into sixteen groups (n = 20), according to the factors: “Ceramic primer application (active or not)”, “Aging simulation (present or not)”, and “Ceramic type (HC, LD, LE or ZLS)”.

Before the surface treatment procedures, all ceramic blocks were immersed in distilled water and ultrasonically cleaned for 5 min (Cristófoli Equipamentos de Biossegurança LTDA, Campo Mourão, Brazil).

-Surface treatments

**Figure 1:**
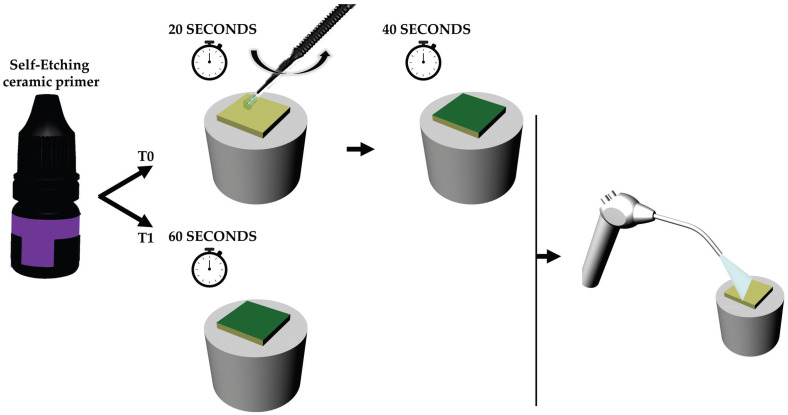
Schematic illustration showing the evaluated surface treatments protocols. T0 was performed following the manufacture’s recommendation using 20 seconds of scrubbing and 40 seconds of reaction time totaling 60 seconds. T1 was performed with 60 seconds of reaction time.

Each group of ceramic material was randomly divided in two other subgroups according to the surface treatment. To evaluate the effect of self-etching ceramic primer active application, two different surface treatments were performed with the same exposing time: In the conventional treatment (T0), the ceramic surface received an active application of self-etching glass ceramic primer (Monobond Etch & Prime, Ivoclar Vivadent, Schaan, Liechtenstein) for 20 s, followed by 40 s of setting. In the experimental treatment (T1), the ceramic surface received self-etching glass ceramic primer (Monobond Etch & Prime, Ivoclar Vivadent, Schaan, Liechtenstein) for 60s of setting (Fig. 1). For both treatments, the samples were then washed with running tap water and dried with an oil-free air jet.

After surface treatment, a cylinder of resin cement (Variolink N, Ivoclar Vivadent, Schaan, Liechtenstein) was made on the ceramics surfaces. A Teflon matrix was used to standardize the adhesive area and height of the cylinder. After fitting the matrix to the surfaces, the resin cement was added to the matrix, light cured for 10 s (1.200 mW/cm2 - Bluephase N, Ivoclar Vivadent, Liechtenstein).

-Resin cement application

After 24 hours, the matrices were removed and half of the samples (block + resin cylinder) were immediately submitted to shear bond strength test, while the other half was submitted to the thermocycling aging protocol. The thermocycling protocol consisted of 10.000 cycles of alternate 30-s baths at 5°C and 55°C, with a 5-s interval between immersions using a thermocycler (Nova Etica, São Paulo, Brazil).

The Shear bond strength test (50 KgF, 0.5mm / min) was carried out in an universal testing machine (DL-1000, EMIC, São José dos Campos, Brazil). Specimens had the cement / ceramic interface hold perpendicularly to the horizontal plane by a device. The load was applied at the base of the cylinder on the adhesive interface, using an orthodontic wire (0.2 mm diameter) at a speed of 0.5 mm/min and load cell of 50 KgF until fracture of the specimen (13).

The calculation of the bond strength was performed by the formula: R = F / A, where R = adhesive strength (MPa); F = force (N); A = interfacial area (mm). The adhesive area of each ceramic block was defined by the area of a circle using the formula A = πr2, where π = 3.14 and r = 1 mm (radius of the cylinder), resulting in a cross-sectional area of 3.14 mm2 (14).

Analysis of variance (3-way ANOVA) and Tukey’s test (5%) were used to compare data from the groups. Using the OpenEpi website, a power of 95.82% was calculated using a two-sided 95% confidence interval.

## Results

Three-way ANOVA revealed that the factors “Material” (*p* = 0.001), “Surface treatment” (*p* = 0.000) and “Aging” (*p* = 0.000) were statistically significant. In addition, the interactions of the factors were also significant. The interaction of all evaluated factors “Material* Surface Treatment *Aging” was significant (*p* = 0.038) and used to perform the Tukey test grouping comparisons to show the difference between the groups (Table 2). When all experimental groups were compared, the use of. The means (± SD) of shear bond strength and the comparison between the experimental groups are summarized in Table 3.

**Table 2:**
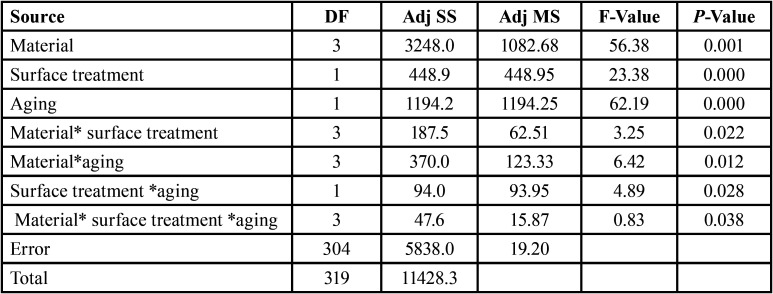
Three-way ANOVA of bond strength according to the factors: material, surface treatment and aging.

**Table 3:**
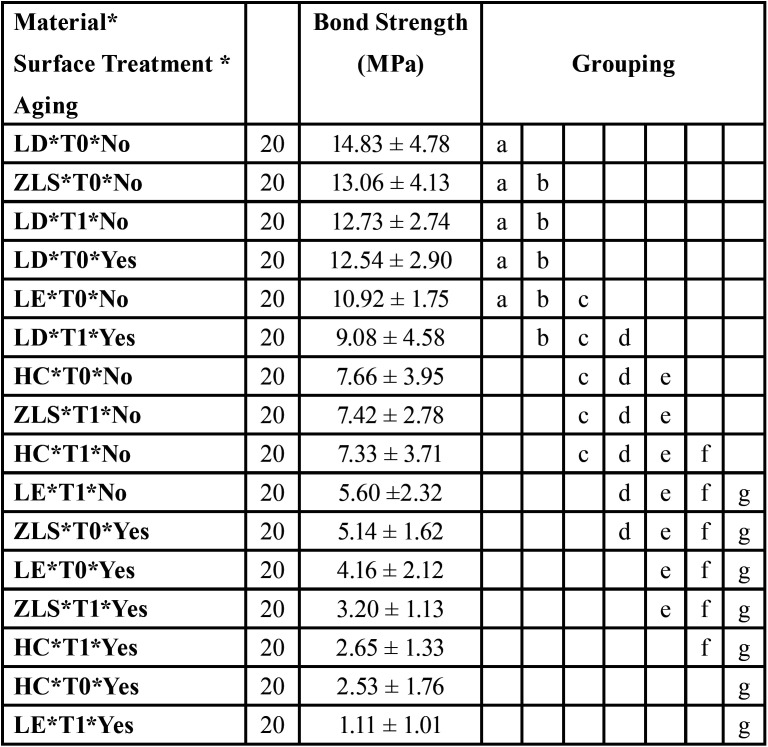
Average bond strength (MPa), standard deviation and TUKEY test (95%) according to the evaluated groups.

According to the results, the aging process has a negative effect to the bond strength for all groups except to Lithium Disilicate based ceramic with active application of the self-etching ceramic primer that has showed bond strength values statistically similar before (14.83 ± 4.78 MPa) and after (12.54 ± 2.90 MPa) aging. However, aged samples without the primer scrubbing showed that the bond strength can be negatively affected even for this ceramic material (decreasing from 12.73 ± 2.74 MPa to 9.08 ± 4.58 MPa). ZLS and LE showed promising results with higher bond strength values for the active application of the self-etching ceramic primer (13.06 ± 4.13 and 10.92 ± 1.75 MPa respectively), however after aging the bond strength value was significantly reduced for both materials (3.20 ± 1.13 and 4.16 ± 2.12 MPa respectively).

When considering the surface treatment with active application, LE and ZLS are statistically similar before (5.60 ±2.32 and 7.42 ± 2.78 respectively) and after aging simulation (1.11 ± 1.01 and 3.20 ± 1.13 MPa respectively). HC showed similar and reduced bond strength values regardless the primer application method (7.66 ± 3.95 MPa for T0 and 7.33 ± 3.71 MPa for T1). This same effect was also noticed after the aging simulation for this restorative material (2.65 ± 1.33 MPa for T0 and 2.53 ± 1.76 MPa for T1).

## Discussion

The purpose of this study was to evaluate the effect of the active application of a self-etching ceramic primer on the bond strength of different CAD/CAM materials. Results presented statistically difference between experimental groups considering the factors interaction. Therefore, the null hypothesis of the study was rejected.

The protocol using a self-etch ceramic primer is a simpler and safer alternative to the conventional protocol with hydrofluoric acid for silica-based ceramics clinical follow-up reporting restorations. This aproach has already a satisfactory esthetic and functional performance, color stability, surface and marginal integrity, and absence of cracks and debonding (15). Therefore, an optimal bond strength can only be achieved when the surface treatment will be properly performed.

A previous study investigated the influence of simplified ceramic surface treatments on shear bond strength of resin-luting cement and lithium disilicate ceramic. The authors indicated that the self-etching ceramic primer is a satisfactory alternative to replace the use of hydrofluoric acid in dental treatments (16). There is also reports that ME&P can be applied as an alternative also for for Leucite based ceramic (10,17). The present study, however suggests that the manufacturers protocol should be followed regardless the restorative material.

According to the literature, when the treating the CAD/CAM materials with self-etching ceramic primer, it is expected the occurrence of the a physicochemical conditioning through a mild etchant (ammonium polifluoride) and a trimethoxypropyl methacrylate for silanization, resulting in a reduced number of defects on the ceramic surface (18). This topographical change is more superficial than those produced by conventional acid etching, which might favor a better fatigue performance (18) and fracture load (17). The same principle can be applied to the bond strength durability when the active application is part of the protocol.

The manufacturer recommends that after being actively applied during 20 seconds, the ME&P must be left in contact with the ceramic surface to let it react and then it must be washed with water. This step is required to remove the acid etchant and reaction byproducts leaving only a thin layer of silane that is chemically bonded to the ceramic surface. It seems that the water washing step is an effective step to remove residuals and so, an additional treatment might not be necessary (19).

A previous study analyzed the effect of different ceramic primers and post-silanization protocols on physicochemical and morphological characteristics of a lithium disilicate glass ceramic. One of the tested surface treatments was the ME&P application, comparing the effect of additional drying with air (30 s) at room temperature than the recommended time. However, the authors did not found influence between these different protocols for the bond strength when self-etching ceramic primer was applied (19). Therefore, we can suggest that the silane exposing time seems to be relevant to the bond strength. In addition, the use of self-etching ceramic primer can produce a lower surface free energy that could be an indicative that the silane molecules might remain effectively bonded to the available hydroxyls on ceramic surface (19).

A previous study evaluated the effect of different scrubbing times (between 5 and 60 seconds) and reaction time (20 and 40 seconds) in two CAD/CAM materials (feldspathic and Lithium disilicate based ceramics) (20). The authors reported that increasing the scrubbing time or the reaction time, a higher glass matrix dissolution will be present, increasing the bond strength for lithium disilicate. For the Feldspathic material, there was no difference between the evaluated protocols (20). According to the authors, the bonding mechanism of the self-etching ceramic primer can be related with the interaction between the ceramic surface ions and functional phosphoric monomers rather than methacrylate silane bonding (20). The present study corroborates with the fact that the scrubbing step is essential for the self-etching silane etching performance.

The results of previous reported accelerated aging tests confirmed that the stability and protective capacity of a silane film to environmental stimuli is dependent on the chemical reactivity, hydrophobicity, extent of network cross-linking and film thickness (21). An additional factor that add for the positive effect of ME&P is the water rinsing and air-drying stage after application, which remove more effectively the loosely bound fractions of γ-methacryloxypropyl trimethoxysilane and bipodal bis-triethoxysilyl ethane (21). However, it seems that this effect can only occur when the scrubbing is performed on the ceramic surface, otherwise the water rising will remove not-reacted silane instead residual components.

The strength and durability of the bond between ceramic and resin cement depends on multiple factors including the type of treatment selected, which is in turn governed by the microstructure of the ceramic material (22). It is also reported that the one-step ceramic primer can be used successfully with the guarantee of excellent bond strength for Leucite based ceramic and reinforced glass ceramics (22,23). However it was also reported that the effect of different surface treatments can affect the biaxial flexure strength, roughness and microstructure of lithium silicate and dislocated reinforced ceramics after cementation and mechanical cycling (24). Similar to the present study, the authors reported the use of self-etching ceramic primer in the ZLS actively applied for 20 s, then reacting for another 40 s. That present study complement these findings showing the ZLS has a good immediate bond strength, however it can be affected by the aging process.

According to an *in vitro* study, the self-etching ceramic primer can be an optimal alternative to Lithium Disilicate based ceramics conditioning, being less hazardous option to treat the ceramic surface (25). However, its use is not recommended for polycrystalline materials such Yttria-stabilized tetragonal zirconia polycrystal (YTZP). Maybe, some interaction between the zirconia present in the ZLS could be the responsible by the low bond strength after aging simulation. However, this hypothesis should be confirmed in further studies.

Comparing the same CAD/CAM materials that were evaluated in the present study, a previous report compared the use of self-etching ceramic primer for 60 s (20 s of application and 40 s of reaction time) and 120 s (40 s of application and 80 s of reaction time) with conventional hydrofluoridric acid etching (26). The authors found that hybrid ceramic had a statistically significantly lower bond strength than lithium disilicate glass-ceramic, whereas the other materials did not differ from the lithium disilicate glass-ceramic (26). In the present study, observing only the results for the conventional treatment (T0) without aging, a similar result can be observed between the present study and the reported literature. In addition to the results and similar materials, the authors defined that future studies exploring different application and reaction times might be useful to come to a definite conclusion about the duration of self-etching ceramic primer (25,26). In this sense, the present study fits this suggestion, demonstrating that the active scrubbing during the self-etching ceramic application is an important step that should not be neglected regardless the CAD/CAM material, especially when long-term bond strength durability is considered.

Several tests can be used in order to verify the bond strength of the ceramic-cement interfaces, such as shear, micro-shear, tensile and micro-tensile. The microtensile bond test is a very useful tool for bond strength investigations, since it can promote a uniform distribution of stresses along the adhesive interface in comparison with the other tests (27). However, pre-testing failure will still occur as a problem for the testing in situations with low bond strength test (27), as well as the sectioning procedures of these tests can induce residual stresses, decreasing the results reliability (28). In this sense, shear bond strength test is widely applied to evaluate the bond strength between the adhesive interfaces of ceramic/resin cement (28-30). Another important aspect to use the shear bond strength test, is the high amount of shear stress concentration that occurs during chewing load incidence in an adhesively luted indirect restoration (31). Therefore, this *in vitro* test can be indicated to simulate one of the predominant stress resultants responsible to increase the adhesive failure risk calculated in dental materials (32).

A restoration in the oral cavity is challenged in many ways: it is subjected to complex occlusal forces, immersed in saliva and exposed to food and beverages with various pH, chemistries, and temperatures (16). However, because this is an *in vitro* study, these reported factors are not present during the aging simulation and are part of the study’s limitations. In addition, there is no incidence of chewing loads in the samples or progressive mechanical fatigue that could affect the bond strength behavior. However, further studies should be carried out to complement the present findings.

## Conclusions

In order to guarantee a durable bond strength, 20 s of active application followed by 40 s of sitting time for the self-etching ceramic primer should be performed when using reinforced-glass ceramics as restorative materials. The lithium disilicate based ceramic demonstrated the highest values of bond strength in comparison with the other evaluated materials, even after the aging simulation.
